# An *ADAM33* Polymorphism Associates with Progression of Preschool Wheeze into Childhood Asthma: A Prospective Case-Control Study with Replication in a Birth Cohort Study

**DOI:** 10.1371/journal.pone.0119349

**Published:** 2015-03-13

**Authors:** Ester M. M. Klaassen, John Penders, Quirijn Jöbsis, Kim D. G. van de Kant, Carel Thijs, Monique Mommers, Constant P. van Schayck, Guillaume van Eys, Gerard H. Koppelman, Edward Dompeling

**Affiliations:** 1 Department of Paediatric Respiratory Medicine, School for Public Health and Primary Care (CAPHRI), Maastricht University Medical Centre (MUMC+), Maastricht, the Netherlands; 2 Department of Epidemiology, CAPHRI, MUMC+, Maastricht, the Netherlands; 3 Department of General Practice, CAPHRI, MUMC+, Maastricht, the Netherlands; 4 Department of Genetics and Cell Biology, Cardiovascular Research Institute (CARIM), MUMC, Maastricht, the Netherlands; 5 Department of Paediatric Pulmonology and Paediatric Allergology, Beatrix Children’s Hospital, Groningen Research Institute for Asthma and COPD (GRIAC), University of Groningen, University Medical Centre Groningen, Groningen, the Netherlands; University of Texas Health Science Center San Antonio Texas, UNITED STATES

## Abstract

**Background:**

The influence of asthma candidate genes on the development from wheeze to asthma in young children still needs to be defined.

**Objective:**

To link genetic variants in asthma candidate genes to progression of wheeze to persistent wheeze into childhood asthma.

**Materials and Methods:**

In a prospective study, children with recurrent wheeze from the ADEM (Asthma DEtection and Monitoring) study were followed until the age of six. At that age a classification (transient wheeze or asthma) was based on symptoms, lung function and medication use. In 198 children the relationship between this classification and 30 polymorphisms in 16 asthma candidate genes was assessed by logistic regression. In case of an association based on a p<0.10, replication analysis was performed in an independent birth cohort study (KOALA study, n = 248 included for the present analysis).

**Results:**

In the ADEM study, the minor alleles of *ADAM33* rs511898 and rs528557 and the *ORMDL3/GSDMB* rs7216389 polymorphisms were negatively associated, whereas the minor alleles of *IL4* rs2243250 and rs2070874 polymorphisms were positively associated with childhood asthma. When replicated in the KOALA study, *ADAM33* rs528557 showed a negative association of the CG/GG-genotype with progression of recurrent wheeze into childhood asthma (0.50 (0.26-0.97) p = 0.04) and no association with preschool wheeze.

**Conclusion:**

Polymorphisms in *ADAM33*, *ORMDL3/GSDMB* and *IL4* were associated with childhood asthma in a group of children with recurrent wheeze. The replication of the negative association of the CG/GG-genotype of rs528557 *ADAM33* with childhood asthma in an independent birth cohort study confirms that a compromised *ADAM33* gene may be implicated in the progression of wheeze into childhood asthma.

## Introduction

Asthma is a common disease in childhood. Twin studies have demonstrated a large contribution of genetic factors to the development of asthma.[1,2] While the cumulative effect of genetic factors may be large, the individual contribution of each factor may be limited. Recently much progress has been made in the field of asthma genetics with the introduction of the genome wide association studies (GWAS). However, these GWAS use general definitions of (doctors diagnosed) asthma, and the specific effect of many candidate genes in relation to the development from wheeze to asthma in young children still needs to be defined.

Asthma is characterized by chronic airway inflammation and airway (hyper-) responsiveness.[3,4] Although asthma starts with wheeze, not all wheezing children will develop asthma. [4,5] It is assumed that at a young age a dysfunction of the maturating immune system at a young age caused by genetic predisposition in combination with environmental triggers, such as environmental tobacco smoke and bacterial infections, can lead to asthma. [6–8] Several asthma candidate genes can be functionally implicated in asthma onset and development. Amongst these are pro-inflammatory genes (*IL4*, *IL5*, *IL8*, *IL13*, *IL33*, *TNFα*), anti-inflammatory genes (*IL10*, *CC16*), genes involved in airway remodelling (*ADAM33*, *PLAUR* and possibly *ORMDL3/GSDMB*), genes involved in the epithelial barrier function (*PCDH1* and possibly *ORMDL3/GSDMB*), genes involved in leukocyte (*ICAM1*) or eosinophil activity (*ORMDL3/GSDMB*) and genes involved in immune modulation (*IL4R*, *LTC4*, *IL1RL1*).

The linkage of genetic variants in asthma candidate genes to progression from early wheeze to persistent wheeze into childhood asthma is expected to result into an increased insight into the pathophysiology of asthma. We therefore aimed to link genetic variants in various asthma candidate genes to progression of early wheeze to persistent wheeze and childhood asthma. Next, we aimed to replicate our findings in an independent birth cohort study.

## Methods

### The ADEM study

#### Study population of the ADEM study

The Asthma DEtection and Monitoring (ADEM) study is a long-term case-control study executed in the Netherlands and is registered at clinicaltrial.gov (NCT 00422747). The aim of this study is to develop a non-invasive instrument for an early asthma diagnosis in children and to study aetiological factors in relation to the early development of asthma.[9] A total of 202 children with recurrent wheeze (≥2 episodes during life according to the International Study of Asthma and Allergies in Childhood (ISAAC) questionnaire)[10] and 50 healthy controls (random selection of children without wheezing episodes during life) at two to four years of age were included. Exclusion criteria were mental retardation, cardiac anomalies, congenital malformations, other diseases of the lungs/airways, Crohn’s disease or rheumatic arthritis, and the inability to perform lung function measurements or exhaled breath collection. For the current analysis, only the children with recurrent wheeze were included.

#### Asthma classification in the ADEM study

At the age of six years a classification (transient wheezer or true asthmatic) was assessed by an experienced paediatrician in the field of respiratory medicine. This classification was based on symptoms, lung function (reversibility to a β_2_-agonist and bronchial hyper-responsiveness) and medication use. In addition to this clinical classification, the classification was assessed by a computer-calculated algorithm as described previously.[11] Bronchial hyper-responsiveness (a 20% fall in forced expiratory volume in one second (FEV_1_) induced by a provocative concentration of histamine <2 mg/ml) and reversibility measurements (bronchodilator reversibility ≥9%) were performed according to the European Respiratory Society guideline.[12] Mismatching cases between the clinical and the computer classification were re-evaluated by the same paediatrician, who was blinded to his previous assessment.

#### DNA isolation and genotyping in the ADEM study

Saliva was collected by Oragene DNA self-collection kits (Oragene, Ottowa, Canada). If children were unable to produce sufficient saliva, buccal cells were obtained. DNA was isolated according to the manufacturer’s protocol. Participants were genotyped for 30 single nucleotide polymorphisms (SNPs) in 16 genes (S1 Table Candidate genes and selected SNPs). Candidate SNPs were selected based on previous association with childhood asthma, and a minor allele frequency of at least 10% (www.hapmap.org phase I, II & III or literature). In total, 27 of the SNPs were analysed by using a mass-spectroscopy based, high-throughput Mass ARRAY iPLEX platform (Sequenom Inc., Hamburg, Germany) using three multiplex genotyping reactions. Sequences were evaluated by ProxSNP and PreXTEND software (http:www.realsnp.com). Sequenom Assay Designer 3.1 software was used to create the different multiplexes. Genotyping was performed according to the iPLEX method. Primer and probe information is provided in S2_Table.docx Primer sequences used for genotyping by Sequenom. Three of the selected SNPs could not be fitted into the multiplex reactions (rs1805010, rs2243250, rs3741240), and therefore these SNPs were determined by Taqman genotyping assays ID C_2769554_10, ID C_16176216_10 and ID C_25473445_10 (Applied Biosystems, California, USA).

### The KOALA Birth Cohort Study

#### Study population of the KOALA Birth Cohort Study

The KOALA study (the Child, Parent and Health: Lifestyle and, Genetic Constitution study) is a prospective study in the Netherlands with the goal of investigating early life risk factors for atopy and asthma.[13] Pregnant healthy women (2,343 women with conventional lifestyle and 491 women with alternative lifestyles) were enrolled at 34 weeks of gestation. Parents were asked to take a buccal swab sample from the child for DNA isolation. From the group of 1,656 children with DNA available, 43 were excluded for congenital conditions like Down’s syndrome and cystic fibrosis or missing baseline data. In the remaining group, follow-up on respiratory complaints until age four years was complete in 1,364 (85%) children. For the main analysis, only the children with recurrent wheeze (≥4 episodes in one questionnaire or at least one episode in multiple questionnaires until four years of age according to the ISAAC questionnaire) and a definitive classification (asthma or transient wheeze) at six to seven years of age were included (n = 248).

#### Asthma diagnosis in the KOALA Birth Cohort Study

Asthma at age six to seven years was defined as ever physician diagnosed asthma with clinical symptoms and/or the use of asthma medication in the last 12 months, adapted from the ISAAC questionnaire.[10] Clinical symptoms were defined as having had at least 1 episode of wheeze or dyspnoea in the last 12 months. The use of asthma medication was defined as regular use (everyday use during at least 2 months or use associated with physical activity) of short-acting β_2_-agonists or the use of inhaled corticosteroids, and medication use according to the Dutch guidelines of treatment of bronchial asthma in children.[14]

#### DNA isolation and genotyping in the KOALA Birth Cohort Study

Parents were asked to collect buccal swabs from their children. Genomic DNA was extracted from these swabs by standard methods.[15] DNA was amplified by using REPLI-g UltraFast technology (Qiagen, Hilden, Germany). Participants were genotyped for five SNPs in three genes that had demonstrated an association with an asthma classification at age six in the ADEM study. Genotyping was performed by Competitive Allele-Specific PCR by using KASPar genotyping chemistry, under contract by LGC Genomics (LGC, Teddington, UK) with extensive quality control as described previously.[16]

### Ethics statement

The ADEM study protocol was approved by the Dutch Central Committee on Research Involving Human Subjects. The KOALA study was approved by the Ethical Committee of the University Hospital of Maastricht. All parents signed informed consent.

### Statistical analysis

IBM SPSS version 20 was used for data analysis (SPSS inc., Chicago IL, USA). Differences in baseline characteristics were evaluated by chi-square test for categorical variables and independent t-tests for continuous parametric variables. The Hardy-Weinberg equilibrium was calculated for each SNP based on a chi-square test and defined deviant in case of a p≥0.05. Furthermore, Linkage disequilibrium (LD) was calculated with Haploview to demonstrate the relationship between SNPs.[17] No attempt was made to analyse the association of haplotypes. Logistic regression with outcome transient wheezer or true asthmatic was performed for each individual SNP. Models were adjusted for sex and exposure to parental smoking and furry pets assessed at time of asthma diagnosis on basis of previous literature and remained in the model irrespective of their statistical significance.[18,19] Both, adjusted and unadjusted results were displayed in the tables. SNPs were tested according to a co-dominant model as this model has been shown to be the most powerful model over the additive, recessive and dominant model to detect associations when the inheritance model is not known.[20] In case the genotype of the two variant alleles was present in <10% of the population of the ADEM study, a dominant model was applied. In case a significant association was observed, all models (co-dominant, dominant and recessive) were applied if the genotype of the two variant alleles was present in ≥10%.

SNPs that demonstrated an association of p<0.10 with the asthma classification in the ADEM study were replicated in the KOALA study and were considered statistically significant when p<0.05. Finally, for SNPs that demonstrated successful replication, we evaluated their association with early wheeze (in order to distinguish it from progression from wheeze to asthma at age four. This was done by logistic regression (adjusted for sex and exposure to parental smoking and furry pets) in the children from the KOALA study with complete follow-up until age four years, with recurrent wheeze as the outcome.

### Power calculation

Due to the specific selection of wheezing children, we anticipated to find large ORs. A group of 146 transient wheezers and 73 asthmatics is sufficient to detect associations with an OR of 2.2 in allele frequency in a dominant model, when assuming the presence of one or more variant alleles of at least 20% in transient wheezers with a power of 0.80 and an alpha of 0.10.

## Results

### The ADEM study

#### Population characteristics

In four children a diagnosis at six years of age could not be assessed due to personal constraints of the parents such as lack of time and interest, leaving 198 children in the current analysis. At the age of six years, 122 children were classified as ‘transient wheezer’ and 76 as ‘true asthmatic’. Characteristics are displayed in Table 1. At the age of six, atopy was higher in the asthmatic group compared to the transient wheeze group.

**Table 1:** Characteristics of the study populations.

	ADEM study		KOALA study	
	Transient wheeze N = 122	True asthma N = 76	Transient wheeze N = 191	True asthma N = 57
Age (years), mean (SD)	6.0 (0.1)	6.0 (0.1)	6.5 (0.6)	6.5 (0.5)
Sex: male / female, in n	63/59	46/30	108/83	37/20
White European descent, %^†^	95	92	97	96
Atopy, %^‡^ ^,^ ^§^	31	47*	79	71
Eczema, %	38	47	39	71*
Exposure to furry pets, %	53	51	49	26*
Exposure to parental smoking, %	36	25	7	2
Parental asthma^§^, %	28	43	28	36

Abbreviations: SD: Standard Deviation; n: number of children; ADEM study: Asthma DEtection and Monitoring study; KOALA study: Kind, Ouders en gezondheid: Aandacht voor Leefstijl en Aanleg study.

† White European descent was defined as at least 3 grandparents from white European (almost all Dutch ancestry) descent.

‡ Atopy was defined as specific IgE concentration against a mixture of inhalant and food allergens of ≥ 0.35 kU/l on the Phadiatop Infant test (Pharmacia, Uppsala, Sweden).

* p<0.05.

^§^based on a limited number of individuals (84 recurrent wheezers, 52 transient wheezers and 27 asthmatics) in the KOALA study.

#### Association of genetic variants with childhood asthma

DNA extraction was successful for all children. All SNPs had a high call rate (92–100%, S1 Table Candidate genes and selected SNPs). No deviation from Hardy-Weinberg equilibrium was observed (p≥0.05) with the exception of *IL1RL1* rs1861245 and *TLR9* rs5743836 (S1 Table Candidate genes and selected SNPs). Three LD blocks were identified (block 1: R^2^ = 0.65 for *ADAM33* rs528557 and rs511898; block 2: R^2^ = 0.56 for *IL4R* rs1805011 and rs1805015, R^2^ = 0.45 for *IL4R* rs1805011 and rs1801275, R^2^ = 0.69 for *IL4R* rs1805015 and rs1801275; block 3: R^2^ = 0.13 for *TLR9* rs187084 and rs5743836).

**Fig 1:**
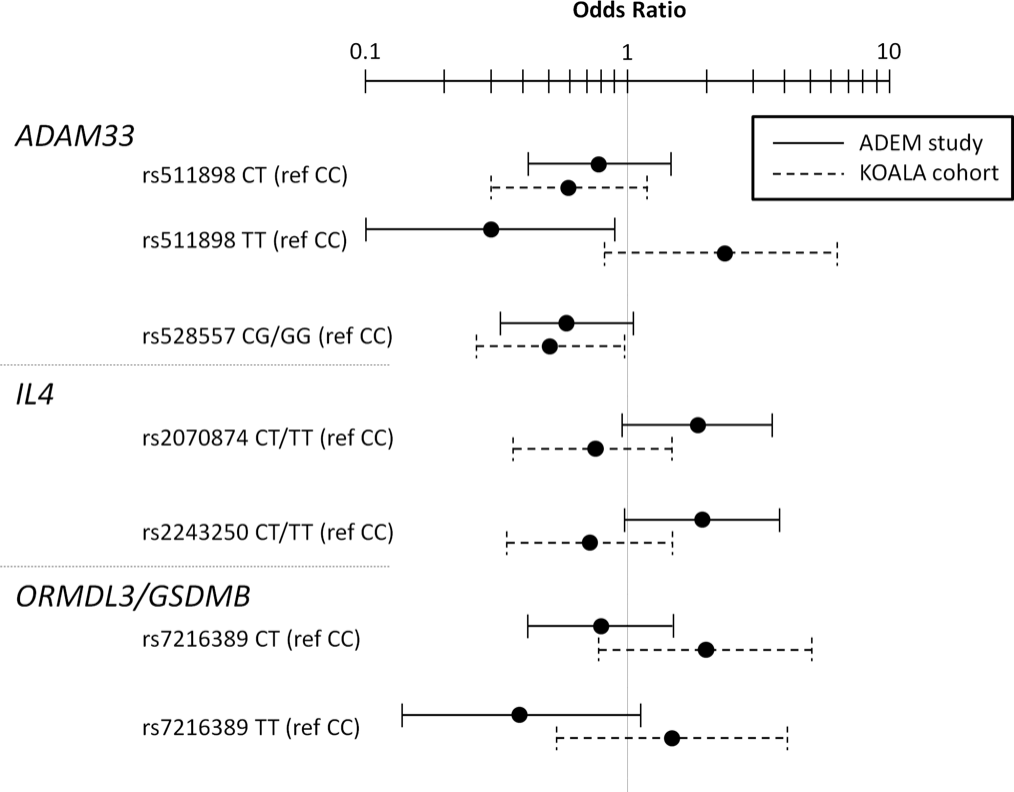
Genetic variants associated with progression from preschool wheeze into childhood asthma in the ADEM study (n = 198) with replication in the KOALA Birth Cohort Study (n = 248)

The TT-genotype of *ADAM33* rs511898 (p = 0.03), the CG/GG-genotype of *ADAM33* rs528557 (p = 0.08) and the TT-genotype of *ORMDL3/GSDMB* rs7216389 (p = 0.08) were negatively associated with childhood asthma. The CT/TT-genotype of *IL4* rs2070874 (p = 0.07) and the CT/TT-genotype of rs2243250 (p = 0.06) were positively associated with childhood asthma (Table 2 and Fig. 1). For *ADAM33* rs511898 and *ORMDL3*/*GSDMB* rs7216389 results of the recessive and dominant model are presented in S3 Table results of the additional model analysis of significant genetic variants in the ADEM study. For *ADAM33* rs528557, *IL4* rs2070874 and *IL4* rs2243250 no alternative models were calculated as the genotype of the two variant alleles was present in <10% of the population. None of the other tested genetic variants demonstrated an association with childhood asthma (S4 Table results for analysis of genetic variants in the ADEM study).

**Table 2:** Genetic variants associated with asthma in the ADEM study.

**Gene**	**SNP**	**allele**	**A/TW (n)**	**OR**	**95%CI**	**p**	**OR** ^**a**^	**95%CI** ^**a**^	**p** ^**a**^
***ADAM33***	**rs511898**	**CC**	32/40	1.00	Reference		1.00	Reference	
		**CT**	38/60	0.81	0.43–1.49	0.49	0.78	0.42–1.47	0.44
		**TT**	5/21	0.30	0.10–0.88	0.03	0.30	0.10–0.90	0.03
	**rs528557**	**CC**	41/49	1.00	Reference		1.00	Reference	
		**CG/GG**	35/73	0.58	0.33–1.04	0.07	0.59	0.33–1.06	0.08
***IL4***	**rs2070874**	**CC**	49/96	1.00	Reference		1.00	Reference	
		**CT/TT**	26/25	2.02	1.05–3.86	0.03	1.86	0.96–3.60	0.07
	**rs2243250**	**CC**	46/93	1.00	Reference		1.00	Reference	
		**CT/TT**	27/25	2.16	1.13–4.13	0.02	1.92	0.98–3.74	0.06
***ORMDL3/GSDMB***	**rs7216389**	**CC**	6/20	1.00	Reference		1.00	Reference	
		**CT**	41/67	0.75	0.40–1.41	0.37	0.80	0.42–1.51	0.49
		**TT**	29/35	0.36	0.13–1.02	0.06	0.39	0.14–1.13	0.08

^a^Adjusted for sex, exposure to parental smoking and furry pets.

Abbreviations: 95%CI: 95% Confidence Interval; A: Asthma; n: number of children; OR: Odds Ratio; p: p-value; TW: Transient Wheeze.

Odds ratios with 95% confidence intervals (horizontal bars) from logistic regression analysis for both the ADEM study and the KOALA study adjusted for sex, exposure to parental smoking and furry pets for those SNPs that demonstrated a significant association with asthma based on a p<0.10 in the ADEM study. Abbreviations: ref; reference category.

### The KOALA Birth Cohort Study

#### Population characteristics

At four years of age, for 1,364 children DNA was available and wheeze classification known (recurrent wheeze versus no recurrent wheeze). Children could only be defined as a child without recurrent wheeze in case all questionnaires until the age of four years were available (n = 1,079). Of the children with recurrent wheeze (n = 285), a definitive classification (asthma or transient wheeze) at the age of six to seven years could not be assessed in 37 children due to missing data. Consequently, a definitive classification (asthma or transient wheeze) was available in 248 children with recurrent wheeze (191 children with transient wheeze and 57 children with asthma). Eczema was significantly more frequent and exposure to furry pets was significantly less frequent in asthmatics compared to transient wheezers at six years of age (Table 1).

#### Replication of associated genetic variants with childhood asthma

All SNPs had a high call-rate (93–96%). No deviation from Hardy-Weinberg equilibrium was observed (p≥0.05). Furthermore, LD was calculated (R^2^ = 0.63 for *ADAM33* rs511898 and rs528557, R^2^ = 0.00 for *IL4* rs2070874 and rs2243250). In the children with recurrent wheeze the CG/GG-genotype of *ADAM33* rs528557 was significantly negatively associated with subsequent childhood asthma when compared to the asthma group (OR (95%CI): 0.50 (0.26–0.97) p = 0.04, Fig. 1 and Table 3). No alternative models were displayed as the genotype of the two variant alleles was present in <10% of the population. When the analysis was restricted to participants with a conventional lifestyle (n = 199), the association did not change (OR (95%CI): 0.46 (0.22–0.94), p = 0.03). *ADAM33* rs511898, *ORMDL3/GSDMB* rs7216389, *IL4* rs2070874 and *IL4* rs2243250 polymorphisms were not associated with childhood asthma in the KOALA study at a 0.05 significance level.

**Table 3:** Replication of associated genetic variants in the KOALA Birth Cohort Study.

**Gene**	**SNP**	**allele**	**A/TW (n)**	**OR**	**95%CI**	**p**	**OR** ^**a**^	**95%CI** ^**a**^	**p** ^**a**^
***ADAM33***	**rs511898**	**CC**	26/76	1.00	Reference		1.00	Reference	
		**CT**	21/91	0.68	0.35–1.29	0.24	0.60	0.30–1.19	0.14
		**TT**	9/12	2.19	0.83–5.80	0.11	2.28	0.82–6.34	0.11
	**rs528557**	**CC**	35/94	1.00	Reference		1.00	Reference	
		**CG/GG**	20/82	0.66	0.35–1.22	0.18	0.50	0.26–0.97	0.04
***IL4***	**rs2070874**	**CC**	41/124	1.00	Reference		1.00	Reference	
		**CT/TT**	14/57	0.74	0.38–1.47	0.39	0.74	0.37–1.50	0.41
	**rs2243250**	**CC**	40/120	1.00	Reference		1.00	Reference	
		**CT/TT**	13/55	0.71	0.35–1.43	0.34	0.72	0.35–1.47	0.36
***ORMDL3 /GSDMB***	**rs7216389**	**CC**	7/39	1.00	Reference		1.00	Reference	
		**CT**	33/89	2.07	0.84–5.07	0.11	2.00	0.78–5.10	0.15
		**TT**	15/54	1.55	0.58–4.15	0.39	1.49	0.54–4.11	0.44

^a^Adjusted for sex, exposure to parental smoking and furry pets. Abbreviations: 95%CI: 95% Confidence Interval; A: Asthma; n: number of children; OR: Odds Ratio; p: p-value; TW: Transient Wheeze.

#### Association between ADAM33 rs528557 and recurrent wheeze at age four

For the replicated gene variant *ADAM33* rs528557 we assessed the role in the presence of recurrent wheeze in the 1,364 children with complete follow-up until age four years, 285 children with recurrent wheeze and 1,079 without recurrent wheeze. The CG/GG-genotype of *ADAM33* rs528557 was not associated with recurrent wheeze at age four (OR (95%CI): 1.03 (0.77–1.39), p = 0.82).

## Discussion

Multiple genetic variants in asthma candidate genes were assessed to determine their relationship with the presence of asthma at six years of age in a group of 202 children with recurrent wheeze at preschool age. We demonstrated association of *ADAM33*, *IL4* and *ORMDL3/GSDMB* gene polymorphisms with childhood asthma. In an independent birth cohort study we were able to replicate the significant negative association of the CG/GG-genotype of *ADAM33* rs528557 with childhood asthma at age six. Since no association was found for this SNP when assessing wheeze at age four, we demonstrated that *ADAM33* is mainly involved in the progression of wheeze into childhood asthma rather than being involved in the presence of recurrent wheeze. The other associations could not be replicated.

*ADAM33* was first reported as a susceptibility gene for asthma and bronchial hyper-responsiveness through genome wide linkage analysis identifying a candidate region on chromosome 20p.[21] *ADAM33* consists of 22 exons which can generate a protein with eight different functional domains.[22] The gene is highly polymorphic, containing more than 70 SNPs with extensive LD. [23–25] Some of the disease-related SNPs encode amino acid changes.[26] Other SNPs are located in the non-coding regions and may affect proliferation of (myo)fibroblasts and smooth muscle and/or inflammation of the airways by alternative splicing and splicing efficiency of messenger RNA turnover or their association is based on linkage with other SNPs.[21,23,26–30] In previous studies, polymorphisms in *ADAM33* have been related to childhood asthma [21,24,31] and impaired lung function in early life in one study.[32] In the present study we found a negative association of the *ADAM33* rs528557 and rs511898 polymorphism with progression of wheeze into childhood asthma. The replication of the negative association of the CG/GG-genotype of rs528557 with childhood asthma in an independent birth cohort study (KOALA study) confirms the relationship of this gene with childhood asthma in (Caucasian) children with recurrent wheeze. Contrary to our findings, the study by van Eerdewegh et al. revealed a positive association of this SNP with asthma in a genome wide scan in 460 Caucasian asthma affected sib-pair families.[21] As the rs528557 polymorphism does not lead to an amino acid change, this conflicting observation might be based on variability of LD between the studied populations. Another explanation might be the pre-selection we applied on children with preschool wheeze resulting in a different stage of asthma development. This was further emphasised by our demonstration that the *ADAM33* rs528557 CG/GG-genotype was not associated with recurrent wheeze at four years of age. Consequently, this SNP is associated with the progression of wheeze into childhood asthma rather than with recurrent preschool wheeze.

*ORMDL3/GSDMB* is mapped to a locus on chromosome 17q12–21, which was first identified in association with childhood asthma through genome-wide analysis.[33] Since then, other studies have confirmed an association of *ORMDL3/GSDMB* with childhood asthma, making it the strongest replicated gene for childhood asthma.[34–37] Its function is still unknown, but it has been suggested that it might have a role in airway remodelling, the epithelial barrier function, or eosinophil trafficking.[38–40] In the present study, the CC-genotype of rs7216389 in *ORMDL3/GSDMB* was demonstrated to be borderline significantly associated with childhood asthma. However, replication failed in the independent birth cohort study. In contrast to our findings, previous studies identified the T-allele of rs7216389 in *ORMDL3/GSDMB* as the childhood asthma risk allele.[34,35,37] Exposure to environmental factors such as tobacco smoke and domestic furry pets, have been demonstrated to modify the relationship between polymorphisms in *ORMDL3/GSDMB* and childhood asthma.[41,42] Remarkable was the difference in prevalence of parental smoking between the ADEM and the KOALA study even though the same definition for passive smoking was used. This is probably due to the different recruitment strategies of the studies. In our analysis correction for sex, exposure of parental smoking and furry pets did not influence our findings. However, we did not assess effect modification in the current study. Furthermore, it might be that unknown environmental influences affect the relationship of *ORMDL3/GSDMB* with asthma, which may explain the difference between our findings and those of others. Moreover, the small sample size of our study might have led to spurious findings due to limited power.

*IL4* maps to a cytokine cluster on chromosome 5q31-q33.[43–45] It is a pro-inflammatory cytokine that is involved in a number of immunoregulatory pathways such as the induction of IgE synthesis by B-cells and differentiation of T-helper-type-2 lymphocytes.[43–46] *IL4* has been linked to asthma phenotypes and atopy in several studies[43,44] including childhood populations.[45,47] In accordance with these findings, we found associations of two SNPs in *IL4* with childhood asthma. This strengthens the suggestion, brought out by previous studies, that children with CT/TT-genotypes for rs2070874 and rs2243250 run an increased risk of developing asthma. Unfortunately, we were unable to replicate these findings in the independent birth cohort study, possibly due to low power.

As seen in the present study, many asthma candidate gene studies are confronted with failure of replication or even opposite findings in independent studies.[32] There are multiple causes for failure of replication. Firstly, asthma is caused by different polymorphisms that do not need to be universal, leading to genetic heterogeneity.[24,29,32,47,48] In addition, the gene effects are small and they may be subject to ethnic diversity and variability in LD between populations, which can lead to population specific results.[43,46] However, the populations of the ADEM study and the KOALA study have, based on region, ethnicity, and genetic origin, similar population characteristics. Furthermore, methodological differences between the studies can influence findings. For example, the definition of asthma used, varies between studies, resulting in different phenotypes. As the asthma classification in the KOALA study differed from the ADEM study, this might be the cause of replication failure in four of the five childhood asthma associated genetic variants. In the ADEM study, the definition of asthma was based on symptoms, lung function features and asthma medication with a high rate of concordance between a doctor diagnosis of asthma and the diagnosis by means of a computer algorithm. In the KOALA study the diagnosis of asthma was based on clinical symptoms and the use of asthma medication which is a universal accepted definition in this type of studies. In addition, it is generally known that environmental influences can affect or even change the direction of underlying associations. Therefore, different environmental conditions between studied populations can be responsible for failure of replication.[29,32,48] In our analysis, we corrected for passive smoking and exposure to furry pets. We cannot fully exclude an influence by other (unknown) environmental factors. Moreover, we concentrated on candidate polymorphisms instead of a complete assessment of all genetic variants due to power restrictions. Although no complete coverage of the variation of each candidate gene was guaranteed as would be the case for tagSNPs, the selected SNPs have previously been proven to be important in association with childhood asthma. Consequently, testing these specific hypotheses, the a priori chance of finding contributing SNPs is large. Thus, we choose not to correct for multiple testing these specific hypotheses. Another reason to ignore multiple testing correction is replication in an independent study which reduces the chance of finding spurious associations. Finally, observed associations can also be caused solely by LD.[29,30,45] Naturally, a significant finding based on chance can also be a cause of replication failure.

In contrast to mentioned limitations, our study has several strengths. The design of the ADEM study enabled us to follow a large group of children with recurrent wheeze at preschool age until the asthma classification at six years of age. Furthermore, our definition of asthma was based on a clinical assessment and a computer-algorithm with re-assessment of inconclusive cases. This is expected to result in a highly accurate classification. Furthermore, replication of found associations was assessed in the independent KOALA study. A limitation of our study might be that we did not correct for multiple testing. However, the use of an independent birth cohort for replication reduced the likelihood of finding associations based on chance.

## Conclusions

In conclusion, we assessed 30 genetic variants in 16 asthma candidate genes in relationship to childhood asthma in a cohort of 202 children with recurrent wheeze. Polymorphisms in *ADAM33* (rs511898 and rs528557) and *ORMDL3/GSDMB* (rs7216389) were negatively associated and polymorphisms in *IL4* (rs2070874 and rs2243250) were positively associated with childhood asthma. In an independent birth cohort we were able to confirm the negative association of *ADAM33* rs528557 CG/GG-genotype with progression of recurrent wheeze into childhood asthma rather than with the presence of wheeze.

## Supporting Information

S1 Table Candidate genes and selected SNPs. Abbreviations: Chr.: chromosome; HWE: Hardy-Weinberg equilibrium;MAF: Minor Allele Frequency; p: p-value; SNP: Single Nucleotide Polymorphism. * based on the ADEM (Asthma DEtection and Monitoring) study.
(DOC)

S2 Table Primer sequences used for genotyping by Sequenom. PCR-1: complementary in direction to the extension primer; PCR-2: in the same direction as the extension primer. The PCR primers contain a 10mer tag that helps to get a balanced amplification in the multiplex PCR and also gets them out of the mass window. The lower case letters in the extension primers are non-template bases that do not affect the annealing temperature but change the mass to allow more efficient multiplexing.
(DOCX)

S3 Table Results of additional model analysis of significant genetic variants in the ADEM study. ^**a**^Adjusted for sex and exposure to parental smoking and furry pets. Abbreviations: 95% CI: 95% Confidence Interval; A: Asthma; n: number of children; OR: Odds Ratio; p: p-value; TW: Transient Wheeze.
(DOCX)

S4 Table Results of analysis for genetic variants in the ADEM study. ^**a**^Adjusted for sex and exposure to parental smoking and furry pets. Abbreviations: 95% CI: 95% Confidence Interval; A: Asthma; n: number of children; OR: Odds Ratio; p: p-value; TW: Transient Wheeze.
(DOC)

## References

[1] Pinto LA, Stein RT, Kabesch M. Impact of genetics in childhood asthma. J Pediatr (Rio J). 2008; 84: S68–75. 10.2223/JPED.1781 18690379

[2] Thomsen SF, van der Sluis S, Kyvik KO, Skytthe A, Skadhauge LR, Backer V. Increase in the heritability of asthma from 1994 to 2003 among adolescent twins. Respir Med 2011;105: 1147–1152. 10.1016/j.rmed.2011.03.007 21450446

[3] Bisgaard H, Szefler S. Prevalence of asthma-like symptoms in young children. Pediatr Pulmonol 2007;42: 723–728. 17598172 10.1002/ppul.20644

[4] Pedersen SE, Hurd SS, Lemanske RF Jr, Becker A, Zar HJ, Sly PD, et al Global strategy for the diagnosis and management of asthma in children 5 years and younger. Pediatr Pulmonol 2011;46: 1–17. 10.1002/ppul.21321 20963782

[5] Savenije OE, Granell R, Caudri D, Koppelman GH, Smit HA, Wijga A, et al Comparison of childhood wheezing phenotypes in 2 birth cohorts: ALSPAC and PIAMA. J Allergy Clin Immunol 2011;127: 1505–1512 e1514. 10.1016/j.jaci.2011.02.002 21411131

[6] Chang JC, Wang L, Chen RF, Liu CA Perinatal gene-gene and gene-environment interactions on IgE production and asthma development. Clin Dev Immunol 2012;2012: 270869 10.1155/2012/270869 22481967 PMC3299317

[7] Bottema RW, Kerkhof M, Reijmerink NE, Thijs C, Smit HA, van Schayck CP, et al Gene-gene interaction in regulatory T-cell function in atopy and asthma development in childhood. J Allergy Clin Immunol 2010;126: 338–346, 346 e331–310. 10.1016/j.jaci.2010.04.024 20599261

[8] Rigoli L, Briuglia S, Caimmi S, Ferrau V, Gallizzi R, Leonardi S, et al Gene-environment interaction in childhood asthma. Int J Immunopathol Pharmacol 2011;24: 41–47. 22032786 10.1177/03946320110240S409

[9] van de Kant KD, Klaassen EM, Jobsis Q, Nijhuis AJ, van Schayck OC, Dompeling E. Early diagnosis of asthma in young children by using non-invasive biomarkers of airway inflammation and early lung function measurements: study protocol of a case-control study. BMC Public Health 2009;9: 210 10.1186/1471-2458-9-210 19563637 PMC2711088

[10] Worldwide variation in prevalence of symptoms of asthma, allergic rhinoconjunctivitis, and atopic eczema: ISAAC. The International Study of Asthma and Allergies in Childhood (ISAAC) Steering Committee. Lancet 1998;351: 1225–1232. 9643741

[11] Klaassen EM, van Kant KD, Jobsis Q, Hovig ST, van Schayck CP, Rijkers GT, et al Symptoms, but not a biomarker response to inhaled corticosteroids, predict asthma in preschool children with recurrent wheeze. Mediators Inflamm 2012;2012: 162571 10.1155/2012/162571 23304059 PMC3523165

[12] Miller MR, Hankinson J, Brusasco V, Burgos F, Casaburi R, Coates A, et al Standardisation of spirometry. Eur Respir J 2005;26: 319–338. 16055882 10.1183/09031936.05.00034805

[13] Kummeling I, Thijs C, Penders J, Snijders BE, Stelma F, Reimerink J, et al Etiology of atopy in infancy: the KOALA Birth Cohort Study. Pediatr Allergy Immunol 2005;16: 679–684. 16343090 10.1111/j.1399-3038.2005.00333.x

[14] Bindels PJ, Grol MH, Ponsioen BP, Salome PL, Wiersma T, Goudswaard AN, et al [Summary of the practice guideline ‘Asthma in children’ (second revision) from the Dutch College of General Practitioners]. Ned Tijdschr Geneeskd 2008;152: 550–555. 18402320

[15] Sambrook J, Russell D (2001) Molecular cloning, a laboratory manual. 3rd ed New York: Cold Spring Harbor Laboratory Press.

[16] Bottema RW, Reijmerink NE, Kerkhof M, Koppelman GH, Stelma FF, Gerritsen J, et al Interleukin 13, CD14, pet and tobacco smoke influence atopy in three Dutch cohorts: the allergenic study. Eur Respir J 2008;32: 593–602. 10.1183/09031936.00162407 18417506

[17] Barrett JC, Fry B, Maller J, Daly MJ. Haploview: analysis and visualization of LD and haplotype maps. Bioinformatics 2005;21: 263–265. 15297300 10.1093/bioinformatics/bth457

[18] Frank E, Harrell J Regression Modeling Strategies. New York: NY: Springer 2001

[19] Steyerberg EW (2009) Clinical Prediction Models. New York: NY: Springer. 2009

[20] Lettre G, Lange C, Hirschhorn JN. Genetic model testing and statistical power in population-based association studies of quantitative traits. Genet Epidemiol. 2007;31: 358–362. 17352422 10.1002/gepi.20217

[21] Van Eerdewegh P, Little RD, Dupuis J, Del Mastro RG, Falls K, Simon J, et al Association of the ADAM33 gene with asthma and bronchial hyperresponsiveness. Nature. 2002;418: 426–430. 12110844 10.1038/nature00878

[22] Holloway JW, Davies DE, Powell R, Haitchi HM, Keith TP, Holgate ST. The discovery and role of ADAM33, a new candidate gene for asthma. Expert Rev Mol Med. 2004;6: 1–12. 10.1017/S1462399404007963 15387895

[23] Ito I, Laporte JD, Fiset PO, Asai K, Yamauchi Y, Martin JG, et al Downregulation of a disintegrin and metalloproteinase 33 by IFN-gamma in human airway smooth muscle cells. J Allergy Clin Immunol. 2007;119: 89–97. 17208589 10.1016/j.jaci.2006.08.038

[24] Noguchi E, Ohtsuki Y, Tokunaga K, Yamaoka-Sageshima M, Ichikawa K, Aoki T, et al ADAM33 polymorphisms are associated with asthma susceptibility in a Japanese population. Clin Exp Allergy. 2006;36: 602–608. 16650044 10.1111/j.1365-2222.2006.02471.x

[25] Lee JH, Park HS, Park SW, Jang AS, Uh ST, Rhim T, et al ADAM33 polymorphism: association with bronchial hyper-responsiveness in Korean asthmatics. Clin Exp Allergy. 2004;34: 860–865. 15196271 10.1111/j.1365-2222.2004.01977.x

[26] Haitchi HM, Powell RM, Shaw TJ, Howarth PH, Wilson SJ, Holgate ST, et al ADAM33 expression in asthmatic airways and human embryonic lungs. Am J Respir Crit Care Med. 2005;171: 958–965. 15709049 10.1164/rccm.200409-1251OC

[27] Foley SC, Mogas AK, Olivenstein R, Fiset PO, Chakir J, Bourbeau J, et al Increased expression of ADAM33 and ADAM8 with disease progression in asthma. J Allergy Clin Immunol. 2007;119: 863–871. 17339047 10.1016/j.jaci.2006.12.665

[28] Kedda MA, Duffy DL, Bradley B, O’Hehir RE, Thompson PJ. ADAM33 haplotypes are associated with asthma in a large Australian population. Eur J Hum Genet. 2006;14: 1027–1036. 16773130 10.1038/sj.ejhg.5201662

[29] Raby BA, Silverman EK, Kwiatkowski DJ, Lange C, Lazarus R, Weiss ST. ADAM33 polymorphisms and phenotype associations in childhood asthma. J Allergy Clin Immunol. 2004;113: 1071–1078. 15208587 10.1016/j.jaci.2004.03.035

[30] Howard TD, Postma DS, Jongepier H, Moore WC, Koppelman GH, Zheng SL, et al Association of a disintegrin and metalloprotease 33 (ADAM33) gene with asthma in ethnically diverse populations. J Allergy Clin Immunol. 2003;112: 717–722. 14564349 10.1016/s0091-6749(03)01939-0

[31] Reijmerink NE, Kerkhof M, Koppelman GH, Gerritsen J, de Jongste JC, Smit HA, et al Smoke exposure interacts with ADAM33 polymorphisms in the development of lung function and hyperresponsiveness. Allergy. 2009;64: 898–904. 10.1111/j.1398-9995.2009.01939.x 19236319

[32] Simpson A, Maniatis N, Jury F, Cakebread JA, Lowe LA, Holgate ST, et al Polymorphisms in a disintegrin and metalloprotease 33 (ADAM33) predict impaired early-life lung function. Am J Respir Crit Care Med. 2005;172: 55–60. 15805180 10.1164/rccm.200412-1708OC

[33] Moffatt MF, Kabesch M, Liang L, Dixon AL, Strachan D, Heath S, et al Genetic variants regulating ORMDL3 expression contribute to the risk of childhood asthma. Nature. 2007;448: 470–473. 17611496 10.1038/nature06014

[34] Sleiman PM, Annaiah K, Imielinski M, Bradfield JP, Kim CE, Frackelton EC, et al ORMDL3 variants associated with asthma susceptibility in North Americans of European ancestry. J Allergy Clin Immunol. 2008;122: 1225–1227. 10.1016/j.jaci.2008.06.041 18760456

[35] Tavendale R, Macgregor DF, Mukhopadhyay S, Palmer CN. A polymorphism controlling ORMDL3 expression is associated with asthma that is poorly controlled by current medications. J Allergy Clin Immunol. 2008;121: 860–863. 10.1016/j.jaci.2008.01.015 18395550

[36] Kavalar MS, Balantic M, Silar M, Kosnik M, Korosec P, Rijavec M. Association of ORMDL3, STAT6 and TBXA2R gene polymorphisms with asthma. Int J Immunogenet. 2012;39: 20–25. 10.1111/j.1744-313X.2011.01051.x 22017802

[37] Wu H, Romieu I, Sienra-Monge JJ, Li H, del Rio-Navarro BE, London SJ. Genetic variation in ORM1-like 3 (ORMDL3) and gasdermin-like (GSDML) and childhood asthma. Allergy. 2009;64: 629–635. 10.1111/j.1398-9995.2008.01912.x 19133921 PMC2697826

[38] Miller M, Tam AB, Cho JY, Doherty TA, Pham A, Khorram N, et al ORMDL3 is an inducible lung epithelial gene regulating metalloproteases, chemokines, OAS, and ATF6. Proc Natl Acad Sci U S A. 2012;109: 16648–16653. 10.1073/pnas.1204151109 23011799 PMC3478632

[39] Holloway JW, Yang IA, Holgate ST. Genetics of allergic disease. J Allergy Clin Immunol. 2010;125: S81–94. 10.1016/j.jaci.2009.10.071 20176270

[40] Ha SG, Ge XN, Bahaie NS, Kang BN, Rao A, Sriramarao P. ORMDL3 promotes eosinophil trafficking and activation via regulation of integrins and CD48. Nat Commun. 2013;4: 2479 10.1038/ncomms3479 24056518 PMC3940275

[41] Bouzigon E, Corda E, Aschard H, Dizier MH, Boland A, Bousquet J, et al Effect of 17q21 variants and smoking exposure in early-onset asthma. N Engl J Med. 2008;359: 1985–1994. 10.1056/NEJMoa0806604 18923164

[42] Blekic M, Kljaic Bukvic B, Aberle N, Marinho S, Hankinson J, Custovic A, et al 17q12–21 and asthma: interactions with early-life environmental exposures. Ann Allergy Asthma Immunol. 2013;110: 347–353 e342. 10.1016/j.anai.2013.01.021 23622005

[43] Basehore MJ, Howard TD, Lange LA, Moore WC, Hawkins GA, Marshik PL, et al A comprehensive evaluation of IL4 variants in ethnically diverse populations: association of total serum IgE levels and asthma in white subjects. J Allergy Clin Immunol. 2004;114: 80–87. 15241348 10.1016/j.jaci.2004.05.035

[44] Beghe B, Barton S, Rorke S, Peng Q, Sayers I, Gaunt T, et al Polymorphisms in the interleukin-4 and interleukin-4 receptor alpha chain genes confer susceptibility to asthma and atopy in a Caucasian population. Clin Exp Allergy. 2003;33: 1111–1117. 12911786 10.1046/j.1365-2222.2003.01731.x

[45] Kabesch M, Tzotcheva I, Carr D, Hofler C, Weiland SK, Fritzsch C, et al A complete screening of the IL4 gene: novel polymorphisms and their association with asthma and IgE in childhood. J Allergy Clin Immunol. 2003;112: 893–898. 14610476 10.1016/j.jaci.2003.08.033

[46] de Faria IC, de Faria EJ, Toro AA, Ribeiro JD, Bertuzzo CS. Association of TGF-beta1, CD14, IL-4, IL-4R and ADAM33 gene polymorphisms with asthma severity in children and adolescents. J Pediatr (Rio J). 2008;84: 203–210. 10.2223/JPED.1783 18425216

[47] Schubert K, von Bonnsdorf H, Burke M, Ahlert I, Braun S, Berner R, et al A comprehensive candidate gene study on bronchial asthma and juvenile idiopathic arthritis. Dis Markers. 2006;22: 127–132. 16788246 10.1155/2006/373620 PMC3851125

[48] Postma DS, Howard T. ADAM33 gene: confirming a gene without linkage. Clin Exp Allergy. 2004;34: 1–3. 14720254 10.1111/j.1365-2222.2004.01845.x

