# Supplementary material for: An ADAM33 Polymorphism Associates with Progression of Preschool Wheeze into Childhood Asthma: A Prospective Case-Control Study with Replication in a Birth Cohort Study
Source: PLoS One. 2015 Mar 13;10(3):e0119349. doi: 10.1371/journal.pone.0119349 (PMC4358930; doi:10.1371/journal.pone.0119349)
Supplement: S1 Table — Abbreviations: Chr.: chromosome; HWE: Hardy-Weinberg equilibrium;MAF: Minor Allele Frequency; p: p-value; SNP: Single Nucleotide Polymorphism. * based on the ADEM (Asthma DEtection and Monitoring) study. (DOC) [file pone.0119349.s001.doc]

*S1_*Table Candidate genes and selected SNPs

| **Gene** | **Chr.** | **SNP** | **Location** | **Major/minor allele** | **MAF***  **(%)** | **HWE* (p)** | **Call rate* (%)** |
| --- | --- | --- | --- | --- | --- | --- | --- |
| *ADAM33* | 20 | rs511898 | Intron | C/T | 38 | 0.41 | 99 |
|  |  | rs528557 | Exon | C/G | 31 | 0.17 | 100 |
|  |  | rs574174 | Intron | C/T | 24 | 0.85 | 99 |
|  |  | rs597980 | Intron | A/G | 51 | 0.48 | 100 |
|  |  | rs2280091 | Exon | A/G | 13 | 0.24 | 100 |
| *CC16* | 11 | rs3741240 | 5’UTR | G/A | 36 | 0.05 | 96 |
| *CD14* | 5 | rs2569190 | Intron | A/G | 51 | 1.00 | 99 |
| *ICAM1* | 19 | rs5498 | Exon | A/G | 37 | 0.02 | 99 |
| *IL1RL1* | 2 | rs1420101 | Intron | C/T | 39 | 0.24 | 99 |
|  |  | rs1861245 | Intron | C/T | 32 | <0.01 | 99 |
| *IL4* | 5 | rs2070874 | 5’UTR | C/T | 14 | 0.93 | 99 |
|  |  | rs2243250 | Promoter | C/T | 15 | 0.37 | 97 |
| *IL4R* | 16 | rs1805010 | Exon | A/G | 46 | 0.34 | 92 |
|  |  | rs1805011 | Exon | A/C | 10 | 0.12 | 100 |
|  |  | rs1805015 | Exon | T/C | 15 | 0.07 | 99 |
|  |  | rs1801275 | Exon | A/G | 20 | 0.48 | 99 |
| *IL5* | 5 | rs2069812 | Promoter | G/A | 34 | 0.27 | 99 |
| *IL8* | 4 | rs2227306 | Intron | C/T | 38 | 0.35 | 99 |
| *IL10* | 1 | rs1800872 | Promoter | G/T | 25 | 0.37 | 100 |
|  |  | rs1800896 | Promoter | T/C | 44 | 0.91 | 99 |
| *IL13* | 1 | rs1800925 | Promoter | C/T | 24 | 0.51 | 99 |
| *IL33* | 5 | rs3939286 | ? | C/T | 28 | 0.29 | 100 |
| *LTC4* | 9 | rs730012 | Promoter | A/C | 28 | 0.11 | 100 |
| *ORMDL3* | 5 | rs7216389 | Intron | T/C | 40 | 0.06 | 100 |
| *PCDH1* | 5 | rs3797054 | Exon | A/G | 35 | 0.26 | 99 |
|  |  | rs3822357 | Exon | C/T | 6 | 0.80 | 100 |
| *PLAUR* | 19 | rs2239372 | Intron | A/G | 46 | 0.14 | 100 |
|  |  | rs4493171 | 5’UTR | C/T | 19 | 0.40 | 99 |
|  |  | rs4803648 | Downstream | T/A | 16 | 0.27 | 99 |
| *TLR2* | 2 | rs3804099 | Intron | T/C | 45 | 0.15 | 99 |
|  |  | rs4696480 | Exon | T/A | 51 | 0.51 | 99 |
| *TLR4* | 9 | rs2737190 | Promoter | A/G | 37 | 0.24 | 99 |
| *TLR9* | 3 | rs187084 | Promoter | A/G | 40 | 0.17 | 99 |
|  |  | rs5743836 | Promoter | A/G | 17 | 0.02 | 99 |
| *TNFα* | 6 | rs1800629 | Promoter | G/A | 16 | 0.86 | 100 |
