# Supplementary material for: An ADAM33 Polymorphism Associates with Progression of Preschool Wheeze into Childhood Asthma: A Prospective Case-Control Study with Replication in a Birth Cohort Study
Source: PLoS One. 2015 Mar 13;10(3):e0119349. doi: 10.1371/journal.pone.0119349 (PMC4358930; doi:10.1371/journal.pone.0119349)
Supplement: S2 Table — PCR-1: complementary in direction to the extension primer; PCR-2: in the same direction as the extension primer. The PCR primers contain a 10mer tag that helps to get a balanced amplification in the multiplex PCR and also gets them out of the mass window. The lower case letters in the extension primers are non-template bases that do not affect the annealing temperature but change the mass to allow more efficient multiplexing. (DOCX) [file pone.0119349.s002.docx]

*S2_Table Primer sequences used for genotyping by Sequenom*

| **Gene** | **SNP** | **Extension primer** | **PCR-1** | **PCR-2** |
| --- | --- | --- | --- | --- |
| *ADAM33* | rs511898 | aAGGCCTGTGAATTCC | ACGTTGGATGAAAATACTGGGACTCGAGGC | ACGTTGGATGATGGGAGTGGGAATGCTGTA |
|  | rs528557 | ccGCTGCCTCTGCTCCCAGG | ACGTTGGATGACCTTCCTGCTGGCCATGCT | ACGTTGGATGAGTCGGTAGCAACACCAGG |
|  | rs597980 | ttACAGGGGCAGCCAGCACC | ACGTTGGATGGGTCACAGAGAACTGGGTTA | ACGTTGGATGTTCACTCCATACCACTGG |
|  | rs574174 | cctcGACTGTCCCCATCCCATC | ACGTTGGATGTGCCCTTGATGATTCCAAGC | ACGTTGGATGGGAACATCACAGGAAATGAC |
|  | rs2280091 | ACGTTGGATGCTGTCCAGTGGCTGTGGG | ACGTTGGATGCCAAAGATGGCCCACACAG | ACGTTGGATGCTGTCCAGTGGCTGTGGG |
| *CD14* | rs2569190 | cctAATCCTTCCTGTTACGG | ACGTTGGATGAGACACAGAACCCTAGATGC | ACGTTGGATGGCAATGAAGGATGTTTCAGG |
| *ICAM* | rs5498 | gagcGCACATTCACGGTCACCT | ACGTTGGATGCTCACAGAGCACATTCACGG | ACGTTGGATGTTGAGGGCACCTACCTCTGT |
| *IL1RL1* | rs1420101 | tcCCATCACAAAGCCTCTCATTA | ACGTTGGATGCGACAACATTTATGTACACC | ACGTTGGATGCTTTAGTAATACTCATTGG |
|  | rs1861245 | GGACATGCATTGACAGA | ACGTTGGATGGGAAAGGTCAGAGGACATGC | ACGTTGGATGTGCCTTCAATGTGTGACAAC |
| *IL4* | rs2070874 | TTAGCTTCTCCTGATAAACTAATTG | ACGTTGGATGTGCATCGTTAGCTTCTCCTG | ACGTTGGATGGAGGTGAGACCCATTAATAG |
| *IL4R* | rs1801275 | tctaGGCCCCCACCAGTGGCTATC | ACGTTGGATGTCCAGCATGGGGCAGCTGCA | ACGTTGGATGACCCTGCTCCACCGCATGTA |
|  | rs1805011 | TCCAGGAGGGAAGGG | ACGTTGGATGAGAGCAGCAGGGATGACTTC | ACGTTGGATGAGGAACAGGCTCTCTGTTAG |
|  | rs1805015 | accgGCTTACCGCAGCTTCAGCAAC | ACGTTGGATGAGAGACGCCCCTCGTCATC | ACGTTGGATGTCTGGGACACGGTGACTGG |
| *IL5* | rs2069812 | gggcgGCTCATGAACAGAATACATA | ACGTTGGATGCCTGCTGCTCATGAACAGAA | ACGTTGGATGCTTGGGCACCTTTCCCATTG |
| *IL8* | rs2227306 | ggGTCATAACTGACAACATTGAAC | ACGTTGGATGACAGTCATAACTGACAACA | ACGTTGGATGCCATGAAGATGTTGATATTG |
| *IL10* | rs1800872 | GACTGGCTTCCTACAG | ACGTTGGATGAAGCAGCCCTTCCATTTTAC | ACGTTGGATGCCTGGAACACATCCTGTGAC |
|  | rs1800896 | tcCCTATCCCTACTTCCCC | ACGTTGGATGCTGGATAGGAGGTCCCTTAC | ACGTTGGATGGACAACACTACTAAGGCTTC |
| *IL13* | rs1800925 | TCCTGCTCTTCCCTC | ACGTTGGATGTGCAGCCATGTCGCCTTTTC | ACGTTGGATGGGGTTTCTGGAGGACTTCTA |
| *IL33* | rs3939286 | ATCCCCATGGTTTGTTGTTG | ACGTTGGATGCAGGAAATTCAGTTCTCCAC | ACGTTGGATGCAGACAGATAACAACAACCC |
| *LTC4* | rs730012 | CCACCTTATCTGTTCCC | ACGTTGGATGACTCCTCCACCCACCTTATC | ACGTTGGATGTTCCGCAGAGGAGGGTTTG |
| *ORMDL3* | rs7216389 | ctccGGGCCGAGTCCATGC | ACGTTGGATGAAGGCCCTTATTAGTGCCTG | ACGTTGGATGAGTATGAAGTGAGGCAACCC |
| *PCDH1* | rs3797054 | GCAATGCTGTAGATCAGCTC | ACGTTGGATGTCTGGAAGAGTCCATAAGGG | ACGTTGGATGAGCCGAGGACTTTGACTCTG |
|  | rs3822357 | AGAGTGTCACTGAGGTC | ACGTTGGATGATGACAACGCACCTGTCTTC | ACGTTGGATGACCAGGCTTGTTGTTTTCCG |
| *PLAUR* | rs2239372 | ATTTACATCCAAAGCCCA | ACGTTGGATGGCTAGCTGTAATTAGGCGAA | ACGTTGGATGGTGGGTGGCCTTGTAGTTAT |
|  | rs4493171 | ggtAGCCTCTCTCCTCAAGATTT | ACGTTGGATGCTCTCAACCGTGATGTTTGG | ACGTTGGATGAGCCCAGGCTTACCTCTTG |
|  | rs4803648 | gCCTTTCTGACCTCAAAACCA | ACGTTGGATGCTCCCAGTATCCTTTCTGAC | ACGTTGGATGAGAAGACACTGGGAGCCAT |
| *TLR2* | rs3804099 | TGAAGGATCAGATGACTTAC | ACGTTGGATGTATGCTGCTTCATATGAAGG | ACGTTGGATGGATCTACAGAGCTATGAGCC |
|  | rs4696480 | AGCCAGATGACCCTC | ACGTTGGATGTCTCACCATGTGATGCTTTC | ACGTTGGATGAGTCCAAGATTGAAGGGCTG |
| *TLR4* | rs2737190 | TTTACACCCAAGTAGACAC | ACGTTGGATGCTCTGAACCACCTCCTCTAC | ACGTTGGATGACCTGTGATGATTAGGGCTG |
| *TLR9* | rs187084 | ATAAAAGATCACTGCCCT | ACGTTGGATGTGCTGGGCACTGTACTGGAT | ACGTTGGATGTATTCCCCTGCTGGAATGTC |
|  | rs5743836 | CCCATGTTCCCTCTGCCTG | ACGTTGGATGTTGGGATGTGCTGTTCCCTC | ACGTTGGATGAGCAGAGACATAATGGAGGC |
| *TNFα* | rs1800629 | GGAGGCTGAACCCCGTCC | ACGTTGGATGGATTTGTGTGTAGGACCCTG | ACGTTGGATGGGTCCCCAAAAGAAATGGAG |
