# Supplementary material for: An ADAM33 Polymorphism Associates with Progression of Preschool Wheeze into Childhood Asthma: A Prospective Case-Control Study with Replication in a Birth Cohort Study
Source: PLoS One. 2015 Mar 13;10(3):e0119349. doi: 10.1371/journal.pone.0119349 (PMC4358930; doi:10.1371/journal.pone.0119349)
Supplement: S3 Table — aAdjusted for sex and exposure to parental smoking and furry pets. Abbreviations: 95% CI: 95% Confidence Interval; A: Asthma; n: number of children; OR: Odds Ratio; p: p-value; TW: Transient Wheeze. (DOCX) [file pone.0119349.s003.docx]

*S3_Table Results of the additional model analysis of significant genetic variants in the ADEM study*

| **Gene** | **SNP** | **allele** | **A/TW (n)** | **OR** | **95%CI** | **p** | **OR^a^** | **95%CI^a^** | **p^a^** |
| --- | --- | --- | --- | --- | --- | --- | --- | --- | --- |
| ***ADAM33***  **rs511898** | **Dominant model** | **CC** | 26/76 | 1.00 | Reference |  | 1.00 | Reference |  |
|  |  | **CT/TT** | 30/103 | 0.67 | 0.37-1.22 | 0.19 | 0.66 | 0.36-1.20 | 0.17 |
|  | **Recessive model** | **CC/CT** | 47/167 | 1.00 | Reference |  | 1.00 | Reference |  |
|  |  | **TT** | 9/12 | 0.34 | 0.12-0.94 | 0.04 | 0.35 | 0.12-0.97 | 0.04 |
| ***ORMDL3 /GSDMB***  **rs7216389** | **Dominant model** | **CC** | 7/39 | 1.00 | Reference |  | 1.00 | Reference |  |
|  |  | **CT/TT** | 40/128 | 0.66 | 0.36-1.21 | 0.18 | 0.71 | 0.38-1.31 | 0.27 |
|  | **Recessive model** | **CC/CT** | 48/143 | 1.00 | Reference |  | 1.00 | Reference |  |
|  |  | **TT** | 15/54 | 0.43 | 0.16-1.13 | 0.09 | 0.45 | 0.17-1.20 | 0.11 |
