# Supplementary material for: An ADAM33 Polymorphism Associates with Progression of Preschool Wheeze into Childhood Asthma: A Prospective Case-Control Study with Replication in a Birth Cohort Study
Source: PLoS One. 2015 Mar 13;10(3):e0119349. doi: 10.1371/journal.pone.0119349 (PMC4358930; doi:10.1371/journal.pone.0119349)
Supplement: S4 Table — aAdjusted for sex and exposure to parental smoking and furry pets. Abbreviations: 95% CI: 95% Confidence Interval; A: Asthma; n: number of children; OR: Odds Ratio; p: p-value; TW: Transient Wheeze. (DOC) [file pone.0119349.s004.doc]

*S4_Table Results for analysis of genetic variants in the ADEM study*

| **Gene** | **SNP** | **allele** | **A/TW (n)** | **OR** | **95% CI** | **p** | **OR^a^** | **95% CI^a^** | **p^a^** |
| --- | --- | --- | --- | --- | --- | --- | --- | --- | --- |
| ***ADAM33*** | **rs511898** | **CC** | 32/40 | 1.00 | Reference |  | 1.00 | Reference |  |
|  |  | **CT** | 38/60 | 0.81 | 0.43-1.49 | 0.49 | 0.78 | 0.42-1.47 | 0.44 |
|  |  | **TT** | 5/21 | 0.30 | 0.10-0.88 | 0.03 | 0.30 | 0.10-0.90 | 0.03 |
|  | **rs528557** | **CC** | 41/49 | 1.00 | Reference |  | 1.00 | Reference |  |
|  |  | **CG/GG** | 35/73 | 0.58 | 0.33-1.04 | 0.07 | 0.59 | 0.33-1.06 | 0.08 |
|  | **rs574174** | **CC** | 46/67 | 1.00 | Reference |  | 1.00 | Reference |  |
|  |  | **CT/TT** | 29/54 | 0.77 | 0.43-1.39 | 0.39 | 0.81 | 0.44-1.47 | 0.48 |
|  | **rs597980** | **AA** | 20/30 | 1.00 | Reference |  | 1.00 | Reference |  |
|  |  | **AG** | 37/57 | 0.97 | 0.48-1.96 | 0.94 | 1.08 | 0.53-2.21 | 0.84 |
|  |  | **GG** | 19/35 | 0.84 | 0.38-1.86 | 0.66 | 0.86 | 0.38-1.96 | 0.73 |
|  | **rs2280091** | **AA** | 62/90 | 1.00 | Reference |  | 1.00 | Reference |  |
|  |  | **AG/GG** | 14/31 | 0.68 | 0.33-1.38 | 0.28 | 0.66 | 0.32-1.37 | 0.27 |
| ***CC16*** | **rs3741240** | **GG** | 24/48 | 1.00 | Reference |  | 1.00 | Reference |  |
|  |  | **GA/AA** | 47/71 | 1.34 | 0.73-2.48 | 0.35 | 1.38 | 0.74-2.57 | 0.32 |
| ***CD14*** | **rs2569190** | **AA** | 18/30 | 1.00 | Reference |  | 1.00 | Reference |  |
|  |  | **GA** | 38/60 | 1.06 | 0.52-2.15 | 0.88 | 1.06 | 0.51-2.20 | 0.87 |
|  |  | **GG** | 19/31 | 1.06 | 0.47-2.40 | 0.90 | 0.98 | 0.42-2.24 | 0.95 |
| ***ICAM1*** | **rs5498** | **AA** | 31/40 | 1.00 | Reference |  | 1.00 | Reference |  |
|  |  | **AG/GG** | 44/81 | 0.68 | 0.38-1.24 | 0.21 | 0.72 | 0.39-1.31 | 0.28 |
| ***IL1RL1*** | **rs1420101** | **CC** | 34/44 | 1.00 | Reference |  | 1.00 | Reference |  |
|  |  | **CT** | 29/56 | 0.67 | 0.36-1.26 | 0.22 | 0.66 | 0.35-1.25 | 0.20 |
|  |  | **TT** | 12/21 | 0.78 | 0.33-1.81 | 0.56 | 0.74 | 0.31-1.74 | 0.49 |
|  | **rs1861245** | **CC** | 31/43 | 1.00 | Reference |  | 1.00 | Reference |  |
|  |  | **CT/TT** | 44/78 | 0.76 | 0.42-1.38 | 0.37 | 0.79 | 0.43-1.44 | 0.44 |
| ***IL4*** | **rs2070874** | **CC** | 49/96 | 1.00 | Reference |  | 1.00 | Reference |  |
|  |  | **CT/TT** | 26/25 | 2.02 | 1.05-3.86 | 0.03 | 1.86 | 0.96-3.60 | 0.07 |
|  | **rs2243250** | **CC** | 46/93 | 1.00 | Reference |  | 1.00 | Reference |  |
|  |  | **CT/TT** | 27/25 | 2.16 | 1.13-4.13 | 0.02 | 1.92 | 0.98-3.74 | 0.06 |
| ***IL4R*** | **rs1805010** | **AA** | 16/33 | 1.00 | Reference |  | 1.00 | Reference |  |
|  |  | **AG** | 40/57 | 1.40 | 0.68-2.89 | 0.36 | 1.44 | 0.69-3.02 | 0.33 |
|  |  | **GG** | 14/22 | 1.27 | 0.52-3.13 | 0.60 | 1.23 | 0.49-3.06 | 0.66 |
|  | **rs1805011** | **AA** | 62/97 | 1.00 | Reference |  | 1.00 | Reference |  |
|  |  | **AC/CC** | 14/25 | 0.87 | 0.42-1.80 | 0.70 | 0.88 | 0.42-1.84 | 0.73 |
|  | **rs1805015** | **TT** | 55/85 | 1.00 | Reference |  | 1.00 | Reference |  |
|  |  | **TC/CC** | 20/36 | 0.85 | 0.45-1.62 | 0.62 | 0.86 | 0.45-1.66 | 0.66 |
|  | **rs1801275** | **AA** | 49/76 | 1.00 | Reference |  | 1.00 | Reference |  |
|  |  | **AG/GG** | 26/45 | 0.88 | 0.48-1.62 | 0.69 | 0.92 | 0.50-1.70 | 0.79 |
| ***IL5*** | **rs2069812** | **GG** | 32/57 | 1.00 | Reference |  | 1.00 | Reference |  |
|  |  | **GA** | 37/44 | 1.50 | 0.81-2.77 | 0.20 | 1.47 | 0.78-2.75 | 0.24 |
|  |  | **AA** | 6/20 | 0.56 | 0.20-1.55 | 0.27 | 0.52 | 0.19-1.45 | 0.21 |
| ***IL8*** | **rs2227306** | **CC** | 28/44 | 1.00 | Reference |  | 1.00 | Reference |  |
|  |  | **CT** | 40/58 | 1.10 | 0.59-2.06 | 0.76 | 1.16 | 0.62-2.19 | 0.64 |
|  |  | **TT** | 7/18 | 0.61 | 0.23-1.65 | 0.33 | 0.64 | 0.24-1.76 | 0.39 |
| ***IL10*** | **rs1800872** | **GG** | 42/67 | 1.00 | Reference |  | 1.00 | Reference |  |
|  |  | **GT/TT** | 34/55 | 1.00 | 0.56-1.79 | 0.99 | 1.00 | 0.56-1.80 | 1.00 |
|  | **rs1800896** | **TT** | 24/37 | 1.00 | Reference |  | 1.00 | Reference |  |
|  |  | **TC** | 37/59 | 0.98 | 0.51-1.90 | 0.96 | 1.03 | 0.53-2.00 | 0.94 |
|  |  | **CC** | 14/25 | 0.86 | 0.38-1.98 | 0.73 | 0.89 | 0.38-2.08 | 0.79 |
| ***IL13*** | **rs1800925** | **CC** | 39/75 | 1.00 | Reference |  | 1.00 | Reference |  |
|  |  | **CT/TT** | 36/45 | 1.52 | 0.85-2.73 | 0.16 | 1.64 | 0.90-2.99 | 0.11 |
| ***IL33*** | **rs3939286** | **CC** | 42/65 | 1.00 | Reference |  | 1.00 | Reference |  |
|  |  | **CT/TT** | 34/57 | 0.91 | 0.51-1.62 | 0.75 | 0.92 | 0.51-1.65 | 0.77 |
| ***LTC4*** | **rs730012** | **AA** | 36/62 | 1.00 | Reference |  | 1.00 | Reference |  |
|  |  | **AC/CC** | 40/60 | 1.17 | 0.66-2.07 | 0.60 | 1.14 | 0.63-2.04 | 0.67 |
| ***ORMDL3*** | **rs7216389** | **TT** | 29/35 | 1.00 | Reference |  | 1.00 | Reference |  |
|  |  | **TC** | 41/67 | 0.75 | 0.40-1.41 | 0.37 | 0.80 | 0.42-1.51 | 0.49 |
|  |  | **CC** | 6/20 | 0.36 | 0.13-1.02 | 0.06 | 0.39 | 0.14-1.13 | 0.08 |
| ***PCDH1*** | **rs3797054** | **AA** | 28/52 | 1.00 | Reference |  | 1.00 | Reference |  |
|  |  | **AG** | 38/58 | 1.24 | 0.67-2.29 | 0.50 | 1.29 | 0.69-2.42 | 0.43 |
|  |  | **GG** | 9/11 | 1.52 | 0.56-4.10 | 0.41 | 1.55 | 0.56-4.32 | 0.40 |
|  | **rs3822357** | **CC** | 66/108 | 1.00 | Reference |  | 1.00 | Reference |  |
|  |  | **CT/TT** | 10/14 | 1.16 | 0.49-2.76 | 0.74 | 1.10 | 0.46-2.65 | 0.83 |
| ***PLAUR*** | **rs2239372** | **AA** | 25/38 | 1.00 | Reference |  | 1.00 | Reference |  |
|  |  | **AG** | 35/53 | 1.02 | 0.53-1.98 | 0.95 | 1.04 | 0.53-2.04 | 0.91 |
|  |  | **GG** | 16/31 | 0.79 | 0.36-1.72 | 0.55 | 0.79 | 0.36-1.75 | 0.56 |
|  | **rs4493171** | **CC** | 50/80 | 1.00 | Reference |  | 1.00 | Reference |  |
|  |  | **CT/TT** | 25/41 | 0.96 | 0.52-1.77 | 0.91 | 0.93 | 0.50-1.73 | 0.82 |
|  | **rs4803648** | **TT** | 55/81 | 1.00 | Reference |  | 1.00 | Reference |  |
|  |  | **TA/AA** | 20/40 | 0.73 | 0.39-1.38 | 0.33 | 0.70 | 0.36-1.33 | 0.27 |
| ***TLR2*** | **rs3804099** | **TT** | 27/37 | 1.00 | Reference |  | 1.00 | Reference |  |
|  |  | **TC** | 32/55 | 0.78 | 0.40-1.51 | 0.45 | 0.73 | 0.37-1.43 | 0.36 |
|  |  | **CC** | 16/29 | 0.74 | 0.33-1.62 | 0.45 | 0.74 | 0.33-1.66 | 0.47 |
|  | **rs4696480** | **TT** | 16/28 | 1.00 | Reference |  | 1.00 | Reference |  |
|  |  | **TA** | 39/63 | 1.10 | 0.53-2.29 | 0.80 | 1.04 | 0.49-2.20 | 0.92 |
|  |  | **AA** | 20/29 | 1.21 | 0.52-2.79 | 0.66 | 1.25 | 0.53-2.93 | 0.61 |
| ***TLR4*** | **rs2737190** | **AA** | 27/54 | 1.00 | Reference |  | 1.00 | Reference |  |
|  |  | **AG** | 36/48 | 1.47 | 0.78-2.77 | 0.23 | 1.57 | 0.81-3.01 | 0.18 |
|  |  | **GG** | 12/19 | 1.24 | 0.53-2.93 | 0.62 | 1.29 | 0.54-3.09 | 0.56 |
| ***TLR9*** | **rs187084** | **AA** | 30/45 | 1.00 | Reference |  | 1.00 | Reference |  |
|  |  | **AG** | 34/51 | 0.98 | 0.52-1.85 | 0.95 | 1.05 | 0.54-2.02 | 0.89 |
|  |  | **GG** | 11/25 | 0.65 | 0.28-1.51 | 0.31 | 0.68 | 0.29-1.59 | 0.37 |
|  | **rs5743836** | **AA** | 50/91 | 1.00 | Reference |  | 1.00 | Reference |  |
|  |  | **AG/GG** | 25/30 | 1.57 | 0.83-2.97 | 0.17 | 1.53 | 0.80-2.91 | 0.20 |
| ***TNFa*** | **rs1800629** | **GG** | 53/85 | 1.00 | Reference |  | 1.00 | Reference |  |
|  |  | **GA/AA** | 23/37 | 0.99 | 0.53-1.84 | 0.96 | 0.99 | 0.52-1.86 | 0.96 |
